# Is Milk Elimination Enough? A Systematic Review and Meta-Analysis of One-Food Elimination Diet in Eosinophilic Esophagitis

**DOI:** 10.3390/medsci14050575

**Published:** 2026-09-16

**Authors:** Stefani Tsaggaridou, Constantinos Pitsios

**Affiliations:** 1 Department of Physiology, School of Medicine, National and Kapodistrian University of Athens, 11527 Athens, Greece; stefanitsaggaridou@gmail.com; 2 Medical School, University of Cyprus, Aglantzia 1056, Nicosia, Cyprus

**Keywords:** eosinophilic esophagitis, milk elimination diet, histological remission

## Abstract

Background/Objectives: Dietary management, particularly through food elimination, has emerged as an effective therapeutic strategy for eosinophilic esophagitis (EoE). In this context, a one-food elimination diet (1-FED) targeting cow’s milk has been proposed as a targeted and less restrictive alternative to broader elimination approaches. The aim of this systematic review and meta-analysis was to systematically assess the effectiveness of 1-FED in achieving histological remission and to compare its efficacy with broader elimination diets in patients with EoE. Methods: A systematic review of the literature was conducted across five databases (PubMed, Scopus, CINAHL, MEDLINE, and ClinicalTrials.gov). The primary endpoint was histological remission (<15 eosinophils/HPF). Comparative studies were pooled using a random-effects meta-analysis. Results: Eight studies met the eligibility criteria for the systematic review, of which seven were included in the meta-analysis. Overall, 156 of 306 patients achieved histological remission following 1-FED, corresponding to a pooled remission rate of 51% (95% CI: 43–60%). Histological remission rates ranged from approximately 34% to 65% across individual studies. Compared with broader elimination diets, 1-FED showed no significant difference in histological remission (OR 1.00, 95% CI 0.64–1.58; *p* = 0.99). Conclusions: Compared to multi-food elimination diets, 1-FED requires a less restrictive dietary pattern, which may offer practical advantages for treatment adherence in routine clinical practice, although this was not systematically or objectively measured in the included studies. The findings lend preliminary support to a step-up dietary strategy, in which 1-FED may be considered as an initial intervention and broader elimination diets are reserved for patients who fail to achieve remission.

## 1. Introduction

Eosinophilic esophagitis (EoE) is a chronic, immune-mediated disease of the esophagus, identified as a distinct clinical entity in the early 1990s [1]. It is characterized by eosinophilic infiltration of the esophageal epithelium, resulting in progressive inflammation and, in some cases, fibrotic remodeling with stricture formation [2,3]. EoE is strongly associated with atopic diseases, may occur at any age and predominantly affects males of Caucasian ethnicity in early adulthood. The prevalence of EoE has increased substantially over the last decades and is currently estimated at approximately 40 cases per 100,000 population [3,4].

The diagnosis of EoE is established by the presence of symptoms of esophageal dysfunction, which vary according to patient age, together with histological evidence of at least 15 eosinophils per high-power field (HPF) in esophageal biopsy specimens [2]. Current management is based on the “3D” approach—Drugs, Diet and Dilation—and treatment should be individualized according to disease phenotype, inflammatory activity, and the presence of fibrostenotic complications [5].

Food allergens are considered the principal drivers of esophageal inflammation in most EoE patients. The efficacy of elimination diets further supports the pathogenic role of food exposure in the disease. Consequently, dietary therapy represents one of the cornerstones of EoE management. Three major dietary approaches have been adopted in clinical practice; elemental diets, empiric food-elimination diets (FEDs), and allergy test-based elimination diets [6]. While elemental diets are highly effective, their poor palatability, low adherence, and impact on quality of life limit their long-term use. Allergy test-based elimination diets have generally demonstrated inferior efficacy compared to empiric elimination diets, which have become the preferred dietary strategy for most patients with EoE [7].

Empiric elimination diets traditionally involve the exclusion of six food groups: cow’s milk, wheat, egg, soy, nuts and seafood. The six-food elimination diet (6FED) has long been considered the standard dietary approach; however, its restrictive nature limits patient adherence and increases nutritional and social burden. These limitations have led to the development of a step-up strategy, beginning with the elimination of fewer food groups and escalating dietary restrictions only in non-responders. Among these approaches, elimination of cow’s milk alone, also referred to as the one-food elimination diet (1-FED), has attracted considerable interest because cow’s milk is consistently identified as the most common dietary trigger of EoE [8,9,10,11].

The aim of the present systematic review and meta-analysis was to critically evaluate the available evidence regarding the effectiveness of a milk elimination diet (1-FED) in patients with EoE, its efficacy compared with broader empiric elimination diets, and its potential role as a first-line dietary strategy in EoE.

## 2. Materials and Methods

### 2.1. Search Strategy and Eligibility Criteria

This systematic review and meta-analysis were designed and conducted in accordance with the Preferred Reporting Items for Systematic Reviews and Meta-Analyses (PRISMA) 2020 statement and followed the Population, Intervention, Comparison, Outcome (PICO) framework for study selection [12]. The protocol for this review was registered in PROSPERO (Registration number: CRD420251131927).

A systematic literature search was performed in PubMed, Scopus, CINAHL (Cumulative Index to Nursing and Allied Health Literature), MEDLINE, and ClinicalTrials.gov for studies published during the previous 20 years. Only articles published in English were considered.

The PICO criteria guided study selection. The population (P) comprised children and adults of any gender with a diagnosis of EoE, confirmed by esophageal biopsies demonstrating ≥15 eosinophils/HPF. The intervention (I) was defined as one food elimination diet (1-FED) targeting cow’s milk and dairy products. Studies evaluating either strict or more liberal forms of 1-FED were eligible, provided that cow’s milk/dairy constituted the only food category targeted for elimination. Other dietary interventions (elemental diets, 6-FED, four-, or two-food elimination diets), no intervention, or pharmacological therapy were examined as comparative interventions (C).

The primary endpoint was histological remission, defined as a peak esophageal eosinophil count < 15 eos/HPF. Although the PROSPERO protocol had specified improvement of clinical symptoms as a co-primary endpoint, this outcome could not be quantitatively synthesized due to inconsistent assessment tools across studies (see Section 3) and is therefore reported alongside the secondary endpoints. Secondary endpoints included improvement of clinical symptoms, changes in health-related quality of life (QoL), adverse effects of the diet, and long-term complications such as esophageal fibrosis or remodeling.

Observational (retrospective and prospective) and interventional studies evaluating the efficacy of a milk-free diet in patients with EoE, including randomized controlled trials and non-randomized intervention studies, investigating the efficacy of a 1-FED in EoE were eligible for inclusion.

Case reports, case series, reviews, opinion articles, editorials, animal and in vitro studies and studies that did not report clinical outcomes related to EoE were excluded. Studies that included other esophageal conditions (such as gastroesophageal reflux, malignancies, or infectious esophagitis), other concomitant therapies, and studies that did not explicitly focus the effect of dairy elimination on EoE were also excluded.

The prespecified protocol excluded studies in which milk elimination was administered in combination with pharmacological therapy. However, during full-text assessment, we distinguished between the initiation of an additional pharmacological treatment concurrently with 1-FED and the continuation of pre-existing PPI therapy. Our intent was to retain studies in which PPI use represented background or pre-existing therapy rather than a newly introduced co-intervention, while excluding studies in which an additional pharmacological treatment was newly initiated concurrently with 1-FED. This represents a deviation from the prespecified PROSPERO protocol and was decided upon to avoid excluding otherwise eligible studies on this basis. Concomitant PPI use in the included studies is described individually in the Results.

The literature search was conducted using targeted keywords and combinations thereof, such as “milk”, “dairy”, “eosinophilic esophagitis”, “EoE”, “food allergy”, “therapy”, “atopy”, “elimination diet”, and “exclusion diet”. Boolean operators (AND, OR) were used to optimize the sensitivity and specificity of the search strategy. After removal of duplicate records, the reference lists of selected articles and relevant reviews were also manually screened to identify additional eligible studies. Searches were conducted in October 2025, consistent with the review timeline registered in PROSPERO. The complete search strategy is provided in Table S1 (Supplementary Materials).

### 2.2. Study Selection, Data Extraction and Analysis

Two reviewers independently screened titles and abstracts, assessed full texts for eligibility, and extracted data using a predefined data extraction form. Disagreements were resolved by consensus.

The methodological quality of non-randomized studies of interventions was assessed using the ROBINS-I tool (Risk of Bias in Non-randomized Studies—of Interventions), whereas randomized controlled trials were evaluated using the Cochrane Risk of Bias 2 (ROB 2) tool [13,14].

### 2.3. Statistical Analysis

The primary meta-analysis estimated the pooled histological remission rate, together with the corresponding 95% confidence interval (CI), while for the second analysis, the odds ratio (OR) with a 95% CI was calculated to compare groups. Heterogeneity across studies was assessed using the chi-squared test (Cochrane Q) and quantified with the I^2^ statistic, with values of 25%, 50%, and 75% generally interpreted as representing low, moderate, and high heterogeneity, respectively [15].

The primary meta-analysis was conducted using R software (version 4.5.1) and applied a random-effects model to estimate the pooled histological remission rate following 1-FED. The comparative meta-analysis was performed using Review Manager (RevMan, version 5.4, Nordic Cochrane Centre, Copenhagen, Denmark), using a random-effects model, to compare histological remission between 1-FED and broader elimination diets (4-FED or 6-FED). Statistical significance was set at *p* < 0.05. For pooling, only the primary histological remission threshold (<15 eos/HPF) was used. Stricter or complete remission thresholds reported by individual studies were summarized descriptively rather than incorporated into the meta-analysis.

## 3. Results

### 3.1. Study Selection and Risk-of-Bias Assessment

In the context of this systematic review, a total of 1284 records were identified through database searching, including Scopus (n = 696), PubMed (n = 410), CINAHL (n = 134), MEDLINE (n = 38), and ClinicalTrials.gov (n = 6). After removing 547 duplicate records (544 using Covidence software and 3 through manual checking), 737 records remained for title and abstract screening. During the initial screening, 571 records were excluded.

The full texts of the remaining 166 reports were assessed for eligibility. Of these, 158 were excluded for the following reasons: review articles (n = 45), systematic reviews or guidelines (n = 17), case reports (n = 10), insufficient data (n = 13), not related to EoE (n = 5), concomitant therapy (n = 6), no milk-elimination diet (n = 55), and not related to dietary treatment (n = 7).

**Figure 1:**
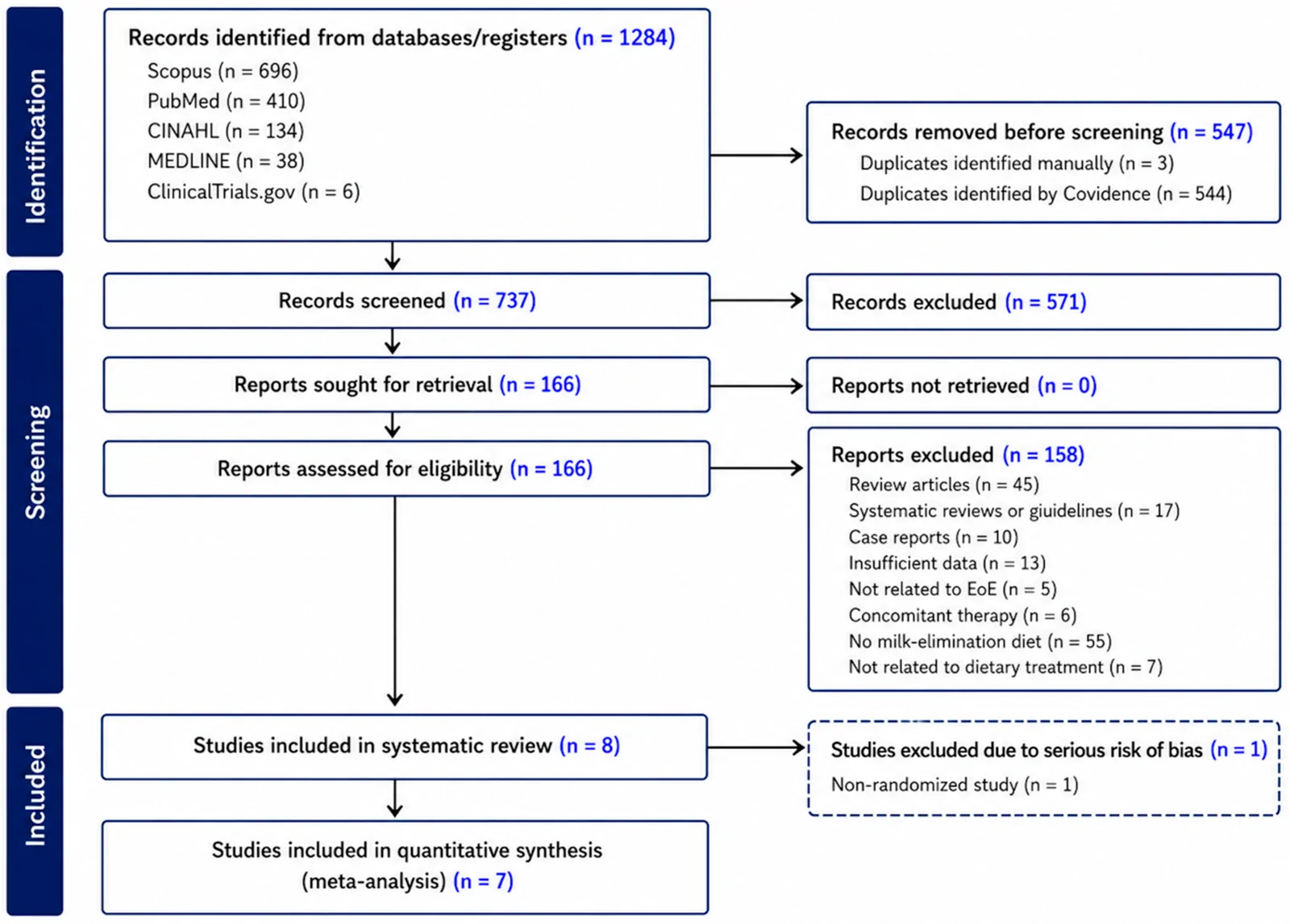
PRISMA 2020 flow diagram of the study selection process.

Overall, eight studies fulfilled the eligibility criteria and were included in the systematic review. Following the risk-of-bias (RoB) assessment, one non-randomized study was judged to have a serious RoB, and was therefore excluded from the quantitative synthesis. Consequently, seven studies were included in the final meta-analysis. The study selection process is summarized in Figure 1.

A domain-specific and overall summary of the ROBINS-I assessment is presented in Table 1. Among the six non-randomized studies, five [10,16,17,18,19] were judged to have a moderate risk of bias according to the ROBINS-I tool [13], while one [20] was judged to have a serious RoB and was consequently excluded from the analysis. This study was judged to have a serious RoB specifically in the domain of confounding (D1), as detailed in Table 1, and was therefore excluded from the quantitative synthesis in accordance with standard guidance to exclude studies with a serious or critical RoB from meta-analytic pooling.

The assessed randomized controlled trials (RCTs) [11,21] provided a clear description of the randomization process and adherence to predefined interventions, while in some cases the evaluators of the histological results were blinded to the intervention. Therefore, these RCTs were judged to have a low risk of bias.

The methodology, principal findings, limitations, and conclusions of the included studies are presented in Table 2.

All seven studies included in the primary meta-analysis defined histological remission using the same threshold (<15 eos/HPF), supporting the validity of pooling this outcome; where individual studies additionally reported stricter or complete remission thresholds, these are noted descriptively in Table 2 but were not used for pooling.

Symptom improvement and QoL outcomes were reported inconsistently across the included studies, using different assessment tools and reporting methods. Therefore, these outcomes could not be quantitatively synthesized and are presented descriptively in Table 2.

Regarding clinical outcomes, symptom improvement was reported in several studies, although assessment methods varied a lot from one study to the next. Where actual numbers were reported, symptom improvement following 1-FED ranged from 61% to 90% [10,18]. In the comparative studies, Kruszewski et al. reported significant symptom improvement with both milk elimination and swallowed fluticasone [17], while Kliewer et al. found no significant difference in symptom-score improvement between the two groups [11]. In the pediatric trial comparing 1-FED with 4-FED, a greater symptom improvement was observed with 4-FED, whereas QoL outcomes were similar between groups [21]. QoL also improved following 1-FED in the studies by Kruszewski et al. and Wechsler et al. [10,17]. Endoscopic outcomes were less consistently reported; in the study of Kliewer et al., no significant difference was found between 1-FED and 6-FED [11].

### 3.2. Overall Effectiveness of a Milk Elimination Diet

**Figure 2:**
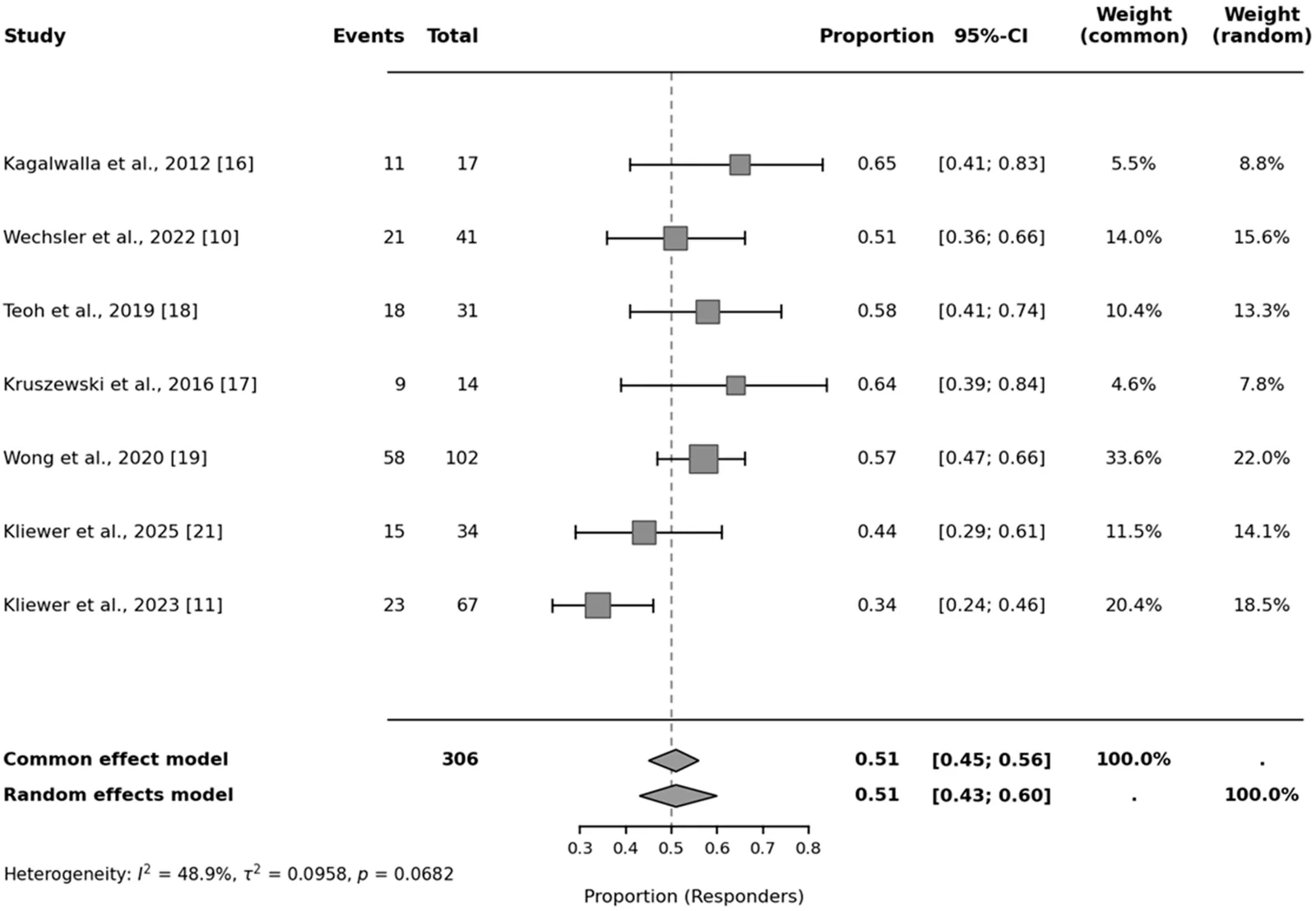
Forest plot of overall response rates across the included studies. Squares represent point estimates for individual studies (size proportional to weight); the dashed line indicates 50% response; diamonds represent pooled estimates from the common- and random-effects models [10,11,16,17,18,19,21].

Histologic remission, defined as fewer than 15 eos/HPF, was the primary outcome of this meta-analysis. Seven studies were included in the quantitative synthesis (Figure 2). Overall, 156 of 306 patients achieved histological remission following a milk elimination diet, corresponding to a pooled remission rate of 51% (95% CI: 43–60%) using a random-effects model. Individual study remission rates ranged from 34% to 65%. Moderate between-study heterogeneity was observed (I^2^ = 48.9%).

### 3.3. Comparative Effectiveness of a Milk Elimination Diet

**Figure 3:**
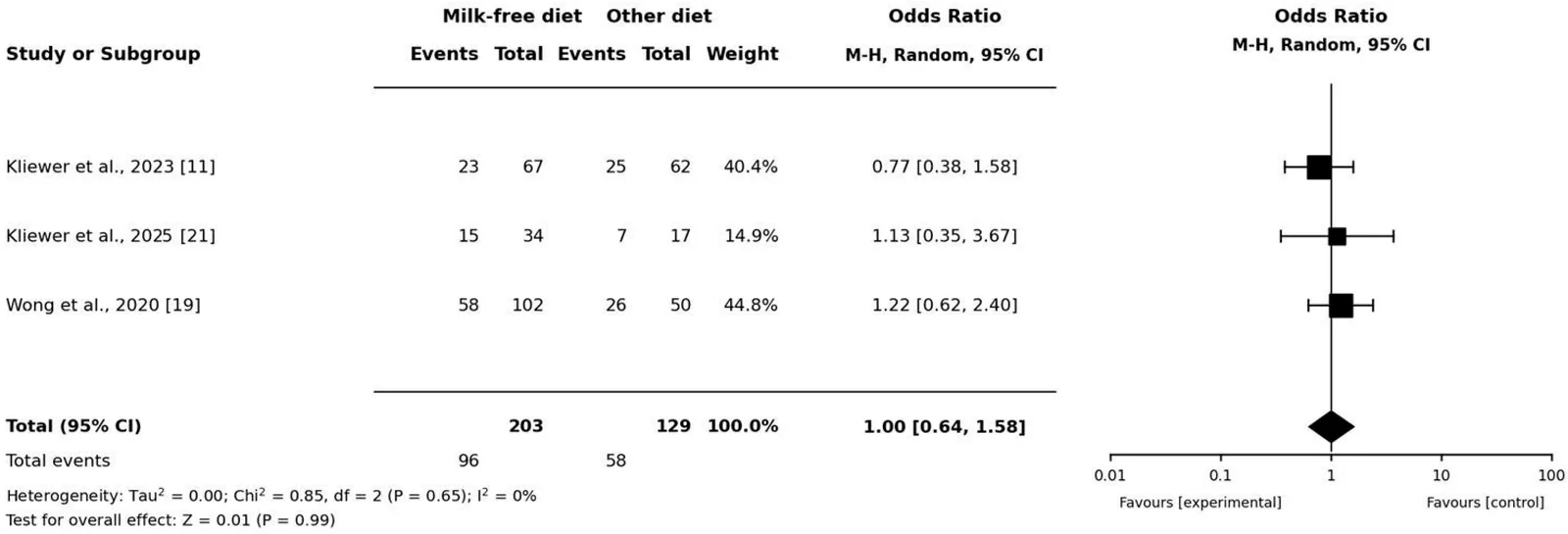
Forest plot comparing one-food elimination diet (1-FED; cow’s milk elimination) with broader empiric food-elimination diets (4-FED or 6-FED). Squares represent the odds ratio for each individual study (size proportional to weight); the vertical line indicates no effect (OR = 1); the diamond represents the pooled odds ratio from the random-effects model [11,19,21].

When studies were grouped according to the type of intervention, three studies (n = 332 participants) compared 1-FED with broader elimination diets (Figure 3) [11,19,21]. The histological response rate was 47.3% (96 of 203) in the 1-FED group and 45.0% (58 of 129) in the comparison diet group. The difference between the groups was not statistically significant (OR 1.00, 95% CI 0.64–1.58; *p* = 0.99), and no heterogeneity was detected (I^2^ = 0%). Publication bias was not assessed because fewer than ten studies were included in the meta-analysis.

## 4. Discussion

Food elimination diets represent one of the therapeutic pillars in the management of EoE. Milk is the most common dietary trigger implicated in the pathogenesis of EoE. A growing body of evidence has documented the central role of cow’s milk in the development of the disease. Cianferoni et al. identified clonally expanded, milk-reactive T helper 2 (TH2) cells with esophageal homing properties, underscoring a targeted, antigen-specific mechanism [22]. Another study demonstrated that immunoglobulin G4 (IgG4) antibodies have been found to form complexes with milk proteins within esophageal tissue, although the functional significance of these complexes remains under investigation [23]. Moreover, exposure to milk proteins was shown to be associated with elevated levels of granzyme B and other mediators involved in the inflammatory cascade [24]. Collectively, these findings support a multifaceted immune response to milk proteins in the pathophysiology of EoE.

The present systematic review and meta-analysis demonstrate that a one-food elimination diet (1-FED) consisting of cow’s milk elimination can induce histological remission in a substantial proportion of patients with EoE. The pooled histological remission rate was 51% (95% CI: 43–60%), with individual study remission rates ranging from 34% to 65%. Moreover, comparative analysis showed no significant advantage of broader empirical elimination diets over milk elimination alone as an initial dietary strategy.

These findings are consistent with those of Grasso et al., who observed histological remission in 50% to 65% of pediatric patients with EoE after adherence to a milk-free diet [25]. They are also in agreement with previous studies identifying cow’s milk as the most frequently implicated dietary trigger of EoE, accounting for approximately 50% to 85% of food-triggered cases [8,26]. Together, these observations reinforce the rationale for considering milk elimination as an initial dietary intervention in patients with EoE.

Most studies evaluating 1-FED have concentrated on pediatric populations, yet evidence indicates that milk elimination can be effective in both children and adults [8,10]. Although cow’s milk is the most frequently implicated dietary trigger in both groups, its role as the sole causative factor appears to differ [10]. In a study including patients from both age groups, histological remission following milk exclusion alone occurred in 33% of children and 18% of adults, suggesting a more multifactorial etiology in the latter [8]. A recent meta-analysis found no statistically significant differences in remission rates between pediatric and adult patients undergoing dietary therapy, indicating that age alone may not determine histological outcomes [27]. Nonetheless, one study reported a higher remission rate in adolescents (~67%) than in children aged 6–12 years (42.9%), although the rate of remission in children < 6 years was 59.3% [19]. In the available studies, remission rates following 1-FED, appeared numerically higher in pediatric patients than in adults (56.5% vs. 34%). However, this finding should be interpreted cautiously because of the small and unequal number of studies across age groups, and no formal statistical comparison between pediatric and adult populations was performed.

In the available comparative studies, no statistically significant difference in histological remission was detected between cow’s milk elimination and more extensive empirical elimination diets. Our findings agree with a recent systematic review and meta-analysis, which reported histological remission rates of 46.4% for 1-FED, 54.7% for 4-FED, and 63.9% for 6-FED [28]. Although remission rates increased with progressively broader dietary restriction, the differences between dietary approaches were relatively modest. Similarly, in the comparative meta-analysis, 1-FED showed no significant difference in histological remission compared with broader empiric food-elimination diets (OR 1.00, 95% CI 0.64–1.58; *p* = 0.99; I^2^ = 0%). However, this finding should be interpreted cautiously because only three comparative studies were available and the comparator interventions included both 4-FED and 6-FED (Figure 3). While 6-FED may achieve higher remission rates, its greater dietary restriction may adversely affect adherence, nutritional adequacy, and quality of life.

The moderate heterogeneity observed in the primary pooled analysis (I^2^ = 48.9%) warrants further consideration, and the pooled remission rate of 51% should be interpreted with caution considering it. The heterogeneity likely reflects differences across the included studies in age population, study design (retrospective vs. prospective vs. randomized), dietary strictness (strict vs. liberal cow’s milk elimination), the comparator used where applicable, duration of follow-up, and the methods used to assess outcomes. These factors should be considered when interpreting the pooled remission estimate, and future studies employing more standardized designs and outcome definitions would help clarify their relative contribution to the observed variability.

The present review and meta-analysis incorporate evidence from both randomized controlled trials and observational studies, covering a broad spectrum of patient populations and clinical settings [10,11,16,17,18,19,21]. This comprehensive inclusion enabled the estimation of an overall histologic remission rate of 51% following a milk elimination diet. Furthermore, this is the first systematic review and meta-analysis specifically evaluating the efficacy of a milk elimination diet as a stand-alone dietary intervention in EoE. Comparative analyses between 1-FED and broader food elimination strategies demonstrated no statistically significant difference in histological remission rates. However, given the limited number of comparative studies and the relatively wide confidence interval, this finding should not be interpreted as evidence of equivalence or non-inferiority. From a practical perspective, initiating dietary treatment with milk elimination alone may avoid unnecessary dietary restrictions for many patients. A less restrictive dietary approach may also facilitate adherence and reduce the nutritional and psychosocial burden associated with broader elimination diets [29]. Overall, the included studies were judged to have a low to moderate risk of bias, with most concerns relating to potential confounding and participant selection in non-randomized studies.

Certain limitations should be acknowledged. Considerable clinical and methodological heterogeneity was present in study design, patient age groups, and definitions of histological remission, potentially influencing the pooled effect estimates. Adherence to milk elimination was assessed inconsistently and was rarely quantified objectively, limiting the ability to evaluate its role in treatment success. Age-specific findings should also be interpreted cautiously because of the small and unequal number of studies involving pediatric and adult populations, which did not allow a meaningful statistical comparison between age groups. In addition, the relatively short duration of follow-up in most studies precludes conclusions regarding the long-term maintenance of histological remission. Furthermore, assessment of symptom improvement and quality of life was inconsistent, preventing a comprehensive evaluation of their relationship with histological remission. Concomitant continuation of PPI therapy was permitted in some studies and may have influenced the observed response to dietary intervention. Finally, despite the encouraging findings, the relatively small number of available studies remains an important limitation. When considered in the context of the GRADE domains, confidence in the available evidence is limited by the moderate risk of bias in most included studies, moderate heterogeneity (I^2^ = 48.9%), and the small number of studies. Confidence in the comparative evidence is further limited by the inclusion of only three comparative studies and the relatively wide confidence interval. Future large-scale randomized trials, with standardized outcome definitions and extended follow-up, are required to determine the long-term efficacy, tolerability, and patient-reported benefits of 1-FED treatment in EoE.

## 5. Conclusions

Based on the currently available evidence, the systematic review and meta-analysis suggest that a milk elimination diet (1-FED) may represent a reasonable initial therapeutic option in selected patients with EoE, particularly when minimizing dietary restriction is a clinical priority. Histological remission rates were substantial, and no statistically significant difference was detected compared with broader dietary interventions in our comparative analysis. The exclusion of a single food group offers potential advantages in terms of treatment adherence, nutritional adequacy, and reduction in social and psychological burden. However, this evidence is derived predominantly from observational studies and a limited number of randomized trials and should be confirmed in larger prospective studies before broader clinical adoption.

These findings lend preliminary support to a step-up therapeutic approach, in which 1-FED is considered as a simple, well-tolerated starting point, with broader elimination diets or pharmacological treatment reserved for patients who fail to achieve remission. Treatment decisions should remain individualized and should involve shared decision-making with patients and families, considering patient characteristics, disease severity, and preferences, and should ideally be supported by a multidisciplinary team. Response to 1-FED should be confirmed through objective reassessment with endoscopy and biopsy rather than symptomatic improvement alone. Further well-designed prospective studies with long-term follow-up are needed to strengthen the evidence and better define the role of 1-FED in the management of eosinophilic esophagitis.

## Figures and Tables

**Table 1:** Risk of bias assessment for non-randomized studies using the ROBINS-I tool.

Study	D1	D2	D3	D4	D5	D6	D7	Overall
Kruszewski, et al. [17]	●	●	●	●	●	●	●	●
Wechsler, et al. [10]	●	●	●	●	●	●	●	●
Teoh, et al. [18]	●	●	●	●	●	●	●	●
Wong, et al. [19]	●	●	●	●	●	●	●	●
Kagalwalla, et al. [16]	●	●	●	●	●	●	●	●
Erwin, et al. [20]	●	●	●	●	●	●	●	●

Domains: D1, bias due to confounding; D2, bias due to selection of participants; D3, bias in classification of interventions; D4, bias due to deviations from intended interventions; D5, bias due to missing data; D6, bias in measurement of outcomes; D7, bias in selection of the reported result. Judgement: ● serious risk of bias, ● moderate risk of bias, ● low risk of bias.

**Table 2:** Summary of studies evaluating the milk elimination diet as a treatment for eosinophilic esophagitis. The methodology, results of the interventions, main findings and limitations of each study are presented.

Study (Year)	Population	Sample Size	Study Design and Intervention	Diet Duration	Concomitant PPI Use	Main Findings	Main Limitations
Kagalwalla (2012) [16]	Children	n = 17	Retrospective study; strict cow’s milk elimination with follow-up endoscopy	Not clearly reported	Not reported	Overall clinical and histological remission in 65%; complete histological remission in 41%	Retrospective design; selection bias
Kruszewski (2016) [17]	Children and adolescents	n = 14 (1-FED) n = 20 (fluticasone), with paired biopsies; 20/24 enrolled	Prospective, comparative trial; cow’s milk elimination vs. swallowed fluticasone	6–8 weeks	Some patients were on concurrent PPI	Histologic remission in 64% with milk elimination vs. 80% with fluticasone; both treatments significantly improved symptoms and QoL (assessed with PedsQL)	
Teoh (2019) [18]	Children	n = 24 strict n = 7 liberal	Retrospective; strict vs. liberal cow’s milk elimination	Not reported	Not reported	Histological remission in 58%; complete remission in 23%; symptom improvement in 90%; higher remission rate with strict elimination (67% vs. 29% with less strict diet)	Small sample; non-randomized; adherence non objectively assessed
Wong (2020) [19]	Children and young adults (<21 years old)	n = 102 (1-FED) n = 50 (6-FED)	Retrospective study comparing dairy-free diet (DFD) with 6-FED	Variable; response analyzed by duration category (<10, 10–12, >12 weeks)	Concurrent PPI use reported, analyzed and was a significant predictor of response	Similar histological response rates between DFD (56.9%) and 6-FED (52.0%); concomitant DFD and PPI therapy had significantly higher response rate than DFD alone (*p* = 0.0177)	Retrospective; heterogeneous management
Wechsler (2022) [10]	Children	n = 41	Prospective observational single-arm study; strict cow’s milk and dairy elimination with follow-up endoscopy after 8–12 weeks	8–12 weeks	PPI-refractory at enrollment; patients on PPI continued it during 1-FED	Histological remission in 51%; symptom improvement in 61%; patient-reported QoL improved	No long-term follow-up
Kliewer (2023) [11]	Adults	n = 67 ((1-FED) n = 62 (6-FED)	Multicenter, randomized trial; milk elimination (1-FED) vs. 6-FED	6 weeks	PPI non-response was an entry criterion; concurrent use during the diet phase not specified	Similar histological remission with 1-FED (34%) and 6-FED (40%); statistically significant higher complete remission with 6-FED; no significant between-group differences in histological, endoscopic, or symptom score improvements	Open-label design; withdrawals; limited power for secondary endpoints
Kliewer (2025) [21]	Children and adolescents	n = 34 ((1-FED) n = 17 (4-FED)	Prospective, multicenter randomized trial; 1FED vs. 4-FED	12 weeks	Not reported	Greater symptom improvement with 4FED than with 1FED; similar histological remission (41% vs. 44%) and QoL outcomes between groups	Small sample size; higher withdrawal rate with 4-FED

## Data Availability

No new data were created or generated in this study. Data sharing is not applicable. All data analyzed in this systematic review and meta-analysis were obtained from the published studies included in the review.

## Supplementary Materials

The following supporting information can be downloaded at https://www.mdpi.com/article/10.3390/medsci14050575/s1. Table S1: Search strategy applied to each database (PubMed/MEDLINE, Scopus, CINAHL, ClinicalTrials.gov).

## Abbreviations

The following abbreviations are used in this manuscript:

EoE	Eosinophilic esophagitis
FED	Food elimination diet
1-FED	One-food elimination diet
4-FED	Four-food elimination diet
6-FED	Six-food elimination diet
HPF	High-power field
RCT	Randomized controlled trial
RoB	Risk of Bias
PRISMA	Preferred Reporting Items for Systematic Reviews and Meta-Analyses
CI	Confidence interval
OR	Odds ratio
DFD	Dairy free diet
PPI	Proton pump inhibitor
PedsQL	Pediatric Quality of Life Inventory
