# Supplementary material for: Is Milk Elimination Enough? A Systematic Review and Meta-Analysis of One-Food Elimination Diet in Eosinophilic Esophagitis
Source: Med Sci (Basel). 2026 Sep 16;14(5):575. doi: 10.3390/medsci14050575 (PMC13609406; doi:10.3390/medsci14050575)
Supplement: Supplementary file 1 [file medsci-14-00575-s001.zip › medsci-4518228-supplementary.pdf]

Supplementary Materials

Table S1. Search strategy applied to each database (PubMed/MEDLINE, Scopus, CINAHL, ClinicalTrials.gov).

Searches were conducted in October 2025, consistent with the review timeline registered in PROSPERO (Registration number: CRD420251131927). The search strategy below applies the same core concepts (eosinophilic esophagitis; milk/dairy; elimination diet and related terms) adapted to each database's specific syntax.

Database	Search string
**PubMed/MEDLINE**	("Eosinophilic Esophagitis"[MeSH] OR "eosinophilic esophagitis"[tiab] OR "EoE"[tiab]) AND ("Milk"[MeSH] OR "milk"[tiab] OR "dairy"[tiab] OR "cow's milk"[tiab]) AND ("elimination diet"[tiab] OR "exclusion diet"[tiab] OR "food allergy"[tiab] OR "atopy"[tiab] OR "therapy"[tiab])
**Scopus**	TITLE-ABS-KEY(("eosinophilic esophagitis" OR "EoE")) AND TITLE-ABS-KEY(("milk" OR "dairy")) AND TITLE-ABS-KEY(("elimination diet" OR "exclusion diet" OR "food allergy" OR "atopy" OR "therapy"))
**CINAHL**	(MH "Eosinophilic Esophagitis" OR TI "eosinophilic esophagitis" OR AB "eosinophilic esophagitis" OR TI "EoE" OR AB "EoE") AND (MH "Milk" OR TI milk OR AB milk OR TI dairy OR AB dairy) AND (TI "elimination diet" OR AB "elimination diet" OR TI "exclusion diet" OR AB "exclusion diet" OR TI "food allergy" OR AB "food allergy" OR TI atopy OR AB atopy OR TI therapy OR AB therapy)
**ClinicalTrials.gov**	Condition: "eosinophilic esophagitis"; Other terms: milk OR dairy OR "elimination diet" OR "exclusion diet"
